# Exploring positive psychology intervention and mindfulness-based intervention in nature: impact on well-being of school students in India

**DOI:** 10.3389/fpubh.2024.1297610

**Published:** 2024-01-31

**Authors:** Raina Chhajer, Nainika Hira

**Affiliations:** ^1^Humanities and Social Sciences Area, Indian Institute of Management, Indore, India; ^2^College of Health, Education, and Human Services, Wright State University, Dayton, OH, United States

**Keywords:** positive psychology intervention, mindfulness-based intervention, nature connectedness, well-being, urban school students, India

## Abstract

**Introduction:**

Enhancing the well-being of urban school students is a growing challenge. The online mode of teaching during and post-pandemic era has increased students’ daily screen time. As they spend more time indoors, they tend to disconnect from nature even more, adversely impacting their well-being. This study aimed to design and execute two well-being interventions—a positive psychology intervention (PPI) and a mindfulness-based intervention (MBI) in natural settings for urban school students in India.

**Methods:**

One hundred eighty participants (aged 17–20) from a senior secondary school were randomly assigned to three groups: PPI, MBI, and a control group (CTR). Participants self-reported their levels of well-being, gratitude, inclusion of nature in self, sense of connectedness, resilience, awareness, perceived stress, and positive and negative emotions using a survey questionnaire at two times—pre- and post-interventions. Repeated-measures ANOVA was employed across time and groups, and *post hoc* analyses for group differences were carried out through the Bonferroni test.

**Results:**

Results indicate that both PPI and MBI interventions, when executed in natural settings, enhance student well-being, gratitude, inclusion of nature in self, sense of connectedness, resilience, awareness, positive emotions and decreased levels of perceived stress, and negative emotions.

**Discussion:**

The study provides valuable insights for school authorities, policymakers, and urban planners to include natural settings in school premises and offer well-being interventions for students to connect with nature consciously.

## Introduction

Now more than ever, school students face multiple challenges to their well-being, warranting immediate attention. In India, students tragically commit suicide at an alarming frequency (1). The distress and vulnerability of students can be attributed to various factors. Mental health stigma in India continues to result in the reluctance to seek professional assistance, and the lack of appropriate mental healthcare adds to it (2). Additionally, excessive academic workload (3), intense competition (4), mounting academic pressures in light of unfulfilled family expectations (5), forced career choices (6), and systemic discrimination (7) contribute extensively to the challenges faced by students in maintaining their mental well-being.

The outbreak of the COVID-19 pandemic has compounded these challenges further. The shift to a virtual mode of education resulted in a significant increase in screen time, which is linked with adverse mental (8) and physical health outcomes (9) for students. Excessive screen time also contributed to sleep difficulties (10), a sedentary lifestyle, and parental fear and control over letting children indulge in outdoor activities. Particularly concerning was that students were also deprived of the opportunity to physically connect with friends, peers, classmates, and relatives during the pandemic (11). Alarmingly, post-pandemic trends indicate that people are dedicating even more time to using connected technology in 2022 than in previous years (12).

While modern lifestyles, technology, and urbanization offer various benefits, it is crucial to recognize the negative consequences they bring, particularly in the context of students’ mental health. These detrimental consequences, coupled with the pandemic, have inadvertently resulted in a phenomenon that is often overlooked but holds significant implications for students’ mental health: *nature disconnection*. Elements such as overcrowding, pollution, reduced access to green spaces, and diminished social support are all effects of urbanization that impact mental health and well-being (13). Nature disconnection has been defined by Beery et al. (14) as “*the lack of awareness or disregard for human identity in material elements and within flows, energy and other nonmaterial elements and values that constitute nature*.”

In India, urban students experience higher levels of mental health challenges, such as loneliness, worry, and suicidal thoughts, as well as issues related to violence, including physical fights and bullying, than their rural counterparts (15). In their article emphasizing the impact of urban environments on the well-being of young individuals, Buttazzoni et al. (16) highlight that urban environments generate substantial noise, which has been linked to increased annoyance and sleep disturbances. Furthermore, such noisy surroundings can diminish social cohesion and the rejuvenating qualities of neighborhoods, contributing to mental health challenges among young individuals, including symptoms such as depression, anxiety, and impaired cognitive function. The Centre for Urban Design and Mental Health (17) has regarded Green, Active, Prosocial, and Safe Places (GAPS) urban design as promoting good mental health. Our ancestors thrived in natural environments throughout history, forming a deep bond with nature. The *biophilia hypothesis* asserts humans have a deep-rooted instinctive affinity for nature and natural environments (18). According to stress recovery theory, as human development has primarily occurred in natural settings, people are physiologically and possibly psychologically better adaptable to natural settings than urban ones (19). This decrease in man’s connection with nature can also be noticed through a more significant cultural shift in the lack of nature-based representations and references in fiction books, song lyrics, and film storylines (20) and Disney animated films for youngsters (21).

According to Louv (22), the term “*nature deficit disorder*” refers to the psychological, physical, and cognitive impacts that can occur when individuals, especially children, are alienated from nature. Nature disconnection can rob young individuals of the possible advantages of interacting with the natural world, encompassing physical, spiritual, mental, and social aspects of their health and well-being (see Table 1) (24). The limited opportunities for student engagement with natural environments have extensive implications, encompassing not only individual well-being but also climate-related apprehensions, attitudes toward the environment, and engagement in conservation activism (25). Conservation efforts worldwide increasingly depend on the younger generation developing significant bonds with nature. In the era of youth-led climate strikes, evidence-based well-being interventions in schools centered on nature can contribute to fostering pro-environmental attitudes and behaviors, fostering a new relationship between pupils, families, and larger communities with nature (26). As per Barrera-Hernández et al. (27), youngsters who feel a stronger connection with nature are also more likely to engage in sustainable actions and report higher happiness levels.

**Table 1:** Benefits of nature connectedness: adapted from Keniger et al. (23).

Benefit	Description	Examples
Psychological well-being	Positive effect on mental processes	Increased self-esteem
		Improved mood
		Reduced anger/frustration
		Psychological well-being
		Reduced anxiety
		Improved behavior
Cognitive	Positive effect on cognitive ability or function	Attentional restoration
		Reduced mental fatigue
		Improved academic performance
		Education/learning opportunities
		Improved ability to perform tasks
		Improved cognitive function in children
		Improved productivity
Physiological	Positive effect on physical function and/or physical health	Stress reduction
		Reduced blood pressure
		Reduced cortisol levels
		Reduced headaches
		Reduced mortality rates from circulatory disease
		Faster healing
		Addiction recovery
		Perceived health/well-being
		Reduced cardiovascular, respiratory disease, and long-term illness
		Reduced occurrence of illness
Social	Positive social effect on an individual, community, or national scale	Facilitated social interaction
		Enables social empowerment
		Reduced crime rates
		Reduced violence
		Enables interracial interaction
		Social cohesion
		Social support
Spiritual	Positive effect on individual religious pursuits or spiritual well-being	Increased inspiration
		Increased spiritual well-being

Thinking about schools as relevant health-promoting settings, positive psychology interventions (PPI), and mindfulness-based interventions (MBI) have emerged as promising well-being interventions. Particularly, MBIs among the school population (28–32), MBIs that infuse direct experiences with nature (33, 34), as well as MBIs in Indian school students (35), have garnered substantial validation. For instance, a recent systematic review and meta-analyses of 25 nature-based MBIs supported the efficacy of these interventions in both open and controlled trials (34). A more recent secondary data analysis of nature-based MBIs concluded that both simulated and real-world natural surroundings improved these outcomes (33).

Similarly, PPIs conducted in a school setting, focusing on positive emotions and positive behaviors, have also received ample support from the literature (36, 37) while having the scope for further investigation in India (38). This is especially relevant for the Indian context: 39% of the Indian population consists of children and youth, often regarded as the nation’s future (39). Positive emotions can significantly and positively influence students’ psychological capital and academic engagement behavior (40). Moreover, PPIs and MBIs make for a great combination as seen by studies that have combined the two intervention approaches (41–43). Ivtzan et al. (42) propose the concept of a “positive mindfulness cycle” in which the reciprocal influence of PPIs and MBIs leads to continuous improvements in Hedonic and Eudaimonic well-being, as MBIs and PPIs consistently reinforce each other, surpassing the individual benefits of practicing mindfulness or PPIs in isolation. Considering this context, this study takes the following theoretical models as a basis for the development of a school-based well-being intervention.

### ART theory

Mindfulness, with its roots in Buddhism, prioritizes the cultivation of a heightened awareness of an individual’s present-moment experience while incorporating an attitude of non-judgmental acceptance toward these experiences (44). While practicing mindfulness, the parasympathetic nervous system gets activated, leading to calm and relaxation, in contrast to the sympathetic nervous system, which is responsible for the body’s “fight-or-flight” response. Additionally, non-judgmental acceptance, which involves changing one’s relationship with one’s own monitored experiences, is essential to improve negative affectivity, stress levels, and overall well-being (45).

The connection between mindfulness and nature becomes increasingly apparent when examining directed attention, a crucial attribute of mindfulness, from the lens of the sensory impact of being in nature (46). Attention restoration theory [see (47)] asserts that nature can replenish attentional deficits and depletions when the natural space provides four qualities: a sense of being away, fascination, the extent of an immersive experience, and compatibility with one’s expectations [Kaplan (47, 48) as cited in Choe and Sheffield (33)]. This theory was derived from literature on esthetics and environmental design preferences [see (49)] and was developed in the mid-1990s, a time period defined by rapid technological advancement and an increasing dominance of indoor entertainment. According to ART, natural environments are conducive to promoting involuntary attention, a less arduous cognitive process, which, in turn, provides a restorative environment that facilitates the recuperation and restoration of directed attention (33). ART posits that individuals can achieve a state of departing from their customary activities and immersing themselves in an environment abundant in natural features and processes. This experiential state is characterized by a psychological detachment from typical demands and routine mental contents, commonly called *being away* (50).

ART is supported by a vast array of research, including a comprehensive meta-analysis of 42 studies which have demonstrated that exposure to nature has a positive impact on working memory, cognitive flexibility, and attentional control, with effects that range from low to moderate in magnitude (51). Kaplan posits that attention restoration processes in natural surroundings are similar to meditation. Such restoration occurs with ease in natural environments without any prior training. In meditation, attention restoration is achieved by focusing on elements such as the breath or sounds. Experienced meditators are believed to accomplish this effortlessly. Individuals who lack experience in meditation and rely on effortful attention regulation during meditation may find support in natural environments where the surroundings facilitate a more effortless restoration of attention (28). Meditating in natural surroundings can temporarily enhance mindfulness, even for individuals such as school students who are not proficient at meditating otherwise (50).

### PERMA model

Positive psychology exercises are activities constructed to foster positive emotions, behaviors, or cognitions (52). The Eco-Existential Positive Psychology perspective, as proposed by Passmore and Howell (53), suggests that our inherent biophilic tendencies can be nurtured through engagement with the natural world. This, in turn, can enhance our overall well-being by providing a means to confront existential anxieties that may arise, including those related to feelings of isolation and happiness (54). The PERMA Model (55) delineates the quintessential constituents of well-being. PERMA is an acronym for Positive Emotion, Engagement, Relationships, Meaning, and Accomplishments.

- *Positive emotions*: This refers to experiencing positive feelings such as joy, love, gratitude, and contentment. Cultivating positive emotions can enhance overall well-being. Numerous studies have demonstrated that exposure to natural environments elicits positive emotions such as joy, awe, and relaxation (56, 57). Nature’s serene and awe-inspiring aspects contribute to an elevated sense of well-being and positive affect.
- *Engagement*: It involves being fully absorbed and immersed in activities that provide a sense of “flow” and fulfillment. Flow and absorption qualities can be achieved by noticing nature and the sensations evoked by natural surroundings.
- *Relationships*: Positive social connections and relationships are crucial for well-being. Nature exposure has been found to enhance connectedness with the natural world and other people (58). Spending time in nature together can strengthen social bonds and foster a sense of connectedness among individuals. Furthermore, nature experiences have been linked to increased prosocial behavior (59) and pro-environmental attitudes (60).
- *Meaning*: A sense of purpose and meaning in life is essential for well-being. Meaning can be cultivated through nature experiences (such as the symbolic value of a tree for understanding resilience) as they provide individuals with a more profound sense of purpose and connection to the natural world. This contemplation often leads to a greater appreciation of nature’s intrinsic value and a recognition of the interdependence between humans and the natural world (61).
- *Accomplishment*: Achieving goals, mastering new skills, and experiencing a sense of accomplishment are essential for well-being. Nature experiences facilitate accomplishment by offering individuals a sense of purpose, goal setting, motivation, and challenge (62) as can be achieved by a “Best Possible Self” (63) exercise in natural surroundings.

To date, the extension of the PERMA (Positive Emotions, Engagement, Relationships, Meaning, and Accomplishment) theory in an intervention conducted in a natural setting remains unexplored, as does a direct comparison between PERMA-based interventions and mindfulness-based interventions in nature, specifically within the context of India. Nature has been associated with various well-being benefits (23), including stress reduction, improved mood, and increased overall life satisfaction. Exploring the extension of the PERMA theory in a natural setting allows for a more comprehensive understanding of well-being, incorporating the potential synergies between positive psychology interventions and nature exposure. If successful, the intervention in a natural setting may have practical applications for interventions outside traditional clinical or controlled environments. This could inform the development of accessible and feasible well-being interventions that leverage natural environments. Considering the widely recognized importance of the natural environment for the well-being of young students, this study aimed to develop, execute, and compare two well-being interventions—PPI and MBI conducted in nature at an urban school in India, with the following research questions:

1. To what extent do PPI and MBI interventions contribute to levels of well-being, gratitude, inclusion of nature in self, sense of connectedness, resilience, awareness, perceived stress, and positive and negative emotions among senior secondary school students in an urban setting?
2. What is the comparative effectiveness of PPI and MBI interventions for senior secondary school students in an urban setting?

## Methods

### Participants

Participants were recruited from an urban senior secondary school in India in November 2022. The eligibility criteria consisted of students in 11 and 12th standards in the age group of 17–20 years. Participants were recruited via purposive sampling as the school premises had a natural outdoor setting to conduct the experiment; 185 students agreed to participate in this study. One hundred eighty eligible participants were randomly divided into three groups using a random number generator, with 60 subjects assigned to each: positive psychology intervention (PPI), mindfulness-based intervention (MBI), and control group (CTR). Table 2 summarizes the sample characteristics for each group, including the gender distribution and mean age. The PPI group had 28 males and 32 females, with a mean age of 17.62 years (SD = 0.49); the MBI group had 28 males and 32 females, with a mean age of 17.50 years (SD = 0.50); and the CTR group had 32 males and 28 females, with a mean age of 17.52 years (SD = 0.50).

**Table 2:** Distribution of sample characteristics for PPI, MBI, and control group.

	Male	Female	Mean age
PPI	28	32	17.62 (0.49)
MBI	28	32	17.50 (0.50)
Control group	32	28	17.52 (0.50)

### Procedure

The Institutional Review Board approved the study procedure (reference number IRB/03/2022-23/HSS). Written informed consent was obtained from the participants. Prior to obtaining written informed consent, an initial establishment of rapport with participants occurred, during which their rights were outlined. Participants were clearly informed of the voluntary nature of their participation, and assurance was provided that all collected information would be utilized solely for research purposes. Additionally, it was emphasized that findings would be presented in an aggregate manner to safeguard individual confidentiality.

Prospective participants received both an information sheet and a consent form before the intervention, ensuring they were well-informed about the study’s objectives and procedures. Following the data collection process, a rigorous coding procedure was implemented, with any identifiable information promptly removed to uphold participant confidentiality. These steps were undertaken to ensure a comprehensive and ethical consent process.

The participants completed the questionnaires in person using a paper–pencil version of the surveys. The data collection was conducted by school teachers external to the study who were blinded to group assignments. Additionally, the research assistant involved in the data entry and coding process was also external to the study. While complete blinding of students was challenging due to their shared school environment, explicit instructions were provided to participants not to share workshop content with their peers until its conclusion. These measures were implemented to minimize bias and enhance the internal validity of the study to the extent possible given the context. Participants were asked to complete self-report measures twice: before the intervention (T1) and immediately after the intervention (T2). Effects were evaluated at post-treatment. The study was conducted in accordance with the Declaration of Helsinki (as revised in 2013) (77).

### Interventions

Participants attended either a PPI or MBI in nature for 5 days, consisting of a 1-h session per day supervised by a qualified facilitator with extensive experience in providing well-being interventions. To ensure consistency in the facilitator’s approach and intervention content across both the PPI and MBI groups, comprehensive measures were implemented. Detailed notes were developed to guide the activities, including scripted elements for most exercises and discussion points. The same facilitator led both groups in the same natural outdoor setting. A control group was also present, which did not receive any intervention.

Participants in PPI and MBI groups performed their given activities in the morning. Interventions were conducted in a natural outdoor setting of the school premises. The green area included a well-managed lawn with seasonal flowers and trees, with an area large enough for the participants to sit in a circle. There were no incentives other than the treatment itself, which was free, including some snacks. The detailed list of activities that were followed in each group is described in Tables 3, 4.

**Table 3:** Overview of the activities of the positive psychology intervention.

Day	Theme	Content	Reference used
1	Positive emotions	Three good thing	Mangan et al. (64)
		*Reflecting on things that one is grateful for and why.*	
		Savoring exercise	Klibert et al. (65)
		*Recalling a moment of joy and fully immersing oneself in the memory.*	
		Joy (Vacation of dreams)	Klibert et al. (65)
		*Imagine a dream vacation, considering the fun, pleasure, and joy it may bring.*	
2	Engagement	Character strengths	Self-Made
		*Sharing a story showcasing one’s strengths while at school.*	
		Creativity	Self-Made
		*Drawing a garden with entities representing loved ones.*	
3	Positive relationships	Loving kindness	Kearney et al. (66), Ivtzan et al. (42)
		*Generating kind intentions through silently repeating positive phrases toward different targets.*	
		Gratitude letter	Kaczmarek et al. (67)
		*Writing a letter expressing gratitude to someone.*	
		Active constructive responding	Gable et al. (68)
		*Learning the communication skill of responding actively and constructively.*	
4	Meaning	Spot the silver lining	Greater Good Science Center (69)
		*Reflecting on a recent frustrating situation and listing three positive aspects of it.*	
		Resilience in self	Self-made
		*Resilience in self through the metaphor of a tree.*	
		Resilience in other models	Self-made
		*Observing and noting resilience in others.*	
5	Accomplishment	Growth mindset	Burnette et al. (70)
		*Imagining challenges faced in academics, compare fixed* versus *growth mindset reactions.*	
		Best possible self	Auyeung et al. (63)
		*Imagining your future self after achieving all your life goals.*	
		WOOP	Saddawi-Konefka et al. (71)
		*Identifying a challenging yet achievable wish and its best possible outcome, acknowledge inner obstacles, and devise an effective plan to overcome them.*	

**Table 4:** Overview of the activities of the mindfulness-based intervention.

Day	Theme	Content	Reference used
1	Mindful awareness	Box breathing	Roisum (72)
		*Inhale for 4 s, hold for 4, exhale for 4, hold for 4.*	
		Mindfulness of sights	Jordan (73), Ballew and Omoto (74)
		*Observing surrounding details and textures and paying attention to colors.*	
		Mindfulness of touch	Adaptation of Jordan (73), Ballew and Omoto (74)
		*Engage senses to feel the shapes and textures of surroundings.*	
2	Mindful walking	Breath awareness	Roisum (72)
		*Focus on breathing and body sensations.*	
		Mindfulness of sound	Adaptation of Roisum (72)
		*Paying close attention to the sounds and pitches around us.*	
		Mindfulness of smells	Adaptation of Roisum (72)
		*Using the sense of smell to pay attention to the fragrance and odors of the surroundings.*	
3	Body-based mindfulness	Body scan relaxation	Roisum (72)
		*Consciously scan each body part, relaxing and releasing tension.*	
		Mindful movement	Roisum (72)
		*Engagement in physical movement with attention to bodily sensations and breath.*	
4	Mindful art	Mindfulness-based Mandalas	Choi et al. (75)
		*Create a Mandala with natural materials available in the natural surroundings while monitoring changes in emotional state*.	
5	Mindfulness of emotion	Emotional regulation	Tara Brach (76)
		*Using the RAIN acronym to be mindful of emotions evoked by natural elements.*	
		Guided imagery	Roisum (72)
		*Visualizing and experiencing a calm and safe personal place.*	

### Measures

At pre-treatment and post-treatment, participants completed the following measures.

#### Well-being

We adopted the 5-item WHO-5 Well-Being Index (78). Respondents had to provide answers using a six-point Likert scale with the following categories: 0 (at no time), 1 (some of the time), 2 (less than half the time), 3 (more than half the time), 4 (most of the time), to 5 (all of the time). The sample item includes, “*I have felt calm and relaxed*.” The reliability calculated using Cronbach’s α was 0.86 (T1) and 0.89 (T2) in this study.

#### Gratitude

We chose the Gratitude Adjective Checklist (GAC) as a brief measure of gratitude (79). It consists of three items or adjectives scored on a Likert scale from 1 (not at all), 2 (a little), 3 (moderately), 4 (quite a bit), to 5 (extremely), with higher scores equating to a higher gratitude. The reliability calculated using Cronbach’s α was 0.79 (T1) and 0.83 (T2) in this study.

#### Nature in self

Schultz’s Nature in Self (INS) is a simple one-item measure with graphical representations that depend on self-report answers. When repeated 1 or 4 weeks later, test–retest correlations for the INS have shown high reliability (80). Each of the seven Venn diagrams in the INS shows two circles labeled “nature” and “self,” with different amounts of overlap. Participants are instructed to circle the illustration most accurately depicting their interaction with the environment (81, 82). The reliability calculated using Cronbach’s α was 0.82 (T1) and 0.84 (T2) in this study.

#### Resilience

The CD-RISC-10, developed by Connor and Davidson (83), was chosen as a measure of resilience for this study. The CD-RISC-10 consists of 10 items. Examples of items on the CD-RISC-10 include “*I can deal with whatever comes my way*” and “*I am not easily discouraged by failure*.” The CD-RISC-10 is scored on a five-point Likert scale, with responses ranging from 0 (not true at all) to 4 (true nearly all the time). The total score is obtained by summing the scores across all 10 items, with higher scores indicating greater resilience. The reliability calculated using Cronbach’s α was 0.80 (T1) and 0.85 (T2).

#### Connection with self, others, and nature

Victorson et al. (84) developed a measure of connection with self, others, and nature. We modified the original questions to make them contextually relevant for school students. Three questions included in the study were as follows: (1) *I felt connected to myself and my ability to achieve goals as a student*; (2) *I felt connected with other students and teachers at school*; and (3) *I felt connected with nature*, scored on a scale of 0–4, where 0 (not at all), 1(very little), 2 (somewhat), 3 (quite a bit), and 4 (a great deal). The reliability calculated using Cronbach’s α was 0.82 (T1) and 0.84 (T2) in this study.

#### Stress

Perceived Stress Scale is a widely used tool used to assess stress, created by Cohen et al. (85). In this study, four items of PSS were used. Some examples include, “*How often have you felt confident about your ability to handle your personal problems?*” and “*How often have you felt that things were going your way?*” A five-point Likert scale is used to score responses, where 0 signifies “never” and 4 indicates “very often.” The reliability calculated using Cronbach’s α was 0.82 (T1) and 0.82 (T2) in this study.

#### Acting with awareness

We adopted three questions from the Five Facet Mindfulness Questionnaire (FFMQ) (86) to measure acting with awareness. We selected the measure of acting with awareness for our study, as it measures the ability to be fully present and engaged in the present moment, rather than distracted by thoughts, worries, or other stimuli. Sample items include, “*I find myself doing things without paying attention.”* A five-point Likert scale is used to score responses, where 1 signifies “never or very rarely true” and 4 indicates “very often or always true.” The reliability calculated using Cronbach’s α was 0.51 (T1) and 0.75 (T2) in this study.

#### Positive and negative affect

PANAS-20 was used to assess both positive mood and negative mood. Respondents rate how much they have experienced each emotion listed on a five-point Likert scale ranging from 1 (very slightly or not at all) to 5 (extremely).

#### Positive affect

The reliability calculated using Cronbach’s α was 0.82 (T1) and 0.86 (T2) in this study. The scale includes 10 items for measuring positive affect. Sample words include *proud* and *interested*.

#### Negative affect

The reliability calculated using Cronbach’s α was 0.80 (T1) and 0.85 (T2) in this study. The scale includes 10 items for measuring negative affect. Sample words include *nervous* and *distressed*.

## Results

Descriptive statistics including means and standard deviations for all measures for PPI, MBI, and CTR groups at the two time points are presented in Table 5. A baseline comparison among the three groups for all study measures was performed, and no significant difference was found. Correlations among study variables are presented in Table 6 at both baseline (above the diagonal) and post-test (below the diagonal). Nature in Self and gratitude had the highest positive correlation (*r* = 0.70, *p* ≤ 0.001). Table 7 presents the differences in study variables from the pre- to post-intervention state for PPI and MBI groups individually. For comparing the differences in study variables from pre- to post-intervention state among the three groups, repeated-measures ANOVA was employed across time and the interaction across time and group. *Post-hoc* analyses for group differences were conducted via the Bonferroni *post-hoc* test, and results are presented in Table 8. The partial eta square (η^2^) or effect size values were considered = 0.20 as small, = 0.50 as medium, and = 0.80 as large as suggested by Cohen (87). All analyses were performed using SPSS Version 27 software, with the significance level set at 95%.

**Table 5:** Baseline and post-test mean scores of all groups on all measures.

	PPI experiment group (*n* = 60)		MBI experiment group (*n* = 60)		Control group (*n* = 60)	
Measures	Baseline Mean (SD)	Post-PPI Intervention (SD)	Baseline Mean (SD)	Post-MBI Intervention (SD)	Baseline Mean (SD)	Post-Mean (SD)
Well-being	2.31 (0.26)	2.83 (0.26)	2.31 (0.25)	2.80 (0.24)	2.33 (0.26)	2.35 (0.28)
Gratitude	2.12 (0.27)	2.86 (0.27)	2.20 (0.35)	2.62 (0.36)	2.10 (0.24)	2.12 (0.25)
Nature in self	2.00 (0.00)	2.87 (0.34)	2.02 (0.13)	2.77 (0.43)	2.03 (0.18)	2.05 (0.22)
Connectivity	2.03 (0.16)	2.70 (0.39)	2.02 (0.12)	2.59 (0.37)	2.04 (0.18)	2.04 (0.18)
Resilience	2.85 (0.20)	2.94 (0.12)	2.73 (0.26)	2.95 (0.13)	2.72 (0.27)	2.72 (0.27)
Stress	2.25 (0.35)	1.20 (0.28)	2.27 (0.34)	1.84 (0.34)	2.28 (0.37)	2.27 (0.36)
Awareness	2.15 (0.36)	2.21 (0.42)	2.09 (0.27)	2.37 (0.45)	2.09 (0.29)	2.09 (0.29)
Positive affect	2.82 (0.20)	2.92 (0.14)	2.75 (0.26)	2.86 (0.22)	2.72 (0.30)	2.71 (0.31)
Negative affect	2.18 (0.21)	2.04 (10)	2.26 (0.25)	2.03 (0.13)	2.27 (0.28)	2.27 (0.28)

**Table 6:** Correlations among all study variables at T1 and T2.

	1	2	3	4	5	6	7	8	9	10	11
1. Age	1	0.05	0.05	−0.04	−0.05	−0.16^*^	0.09	0.07	−0.05	0.01	−0.04
2. Gender	0.05	1	0.01	0.11	−0.04	0.03	−0.05	−0.06^*^	0.15^*^	−0.05^*^	−0.02
3. Well-being	0.05	−0.06	1	0.18^*^	0.14	0.02	0.2^*^	−0.1	−0.07	0.04	0.01
4. Gratitude	0.01	0.02	0.45^**^	1	0.38^**^	0.17^*^	0.09	−0.23^**^	0.06	0.11	−0.02
5. NIS	0.07	−0.03	0.46^**^	0.70^**^	1	0.16^*^	0.07	−0.1	−0.05	0.07	−0.07
6. Connectivity	−0.03	0.02	0.48^**^	0.62^**^	0.64^**^	1	0.06	−0.03	−0.00	0.04	−0.10
7. Resilience	0.09	−0.02	0.40^**^	0.48^**^	0.50^**^	0.48^**^	1	−0.3^**^	−0.13	0.61^**^	0.52^**^
8. Stress	−0.12	−0.06	−0.38^**^	−0.42^**^	−0.34^**^	−0.39^**^	−0.38^**^	1	0.22^**^	−0.35^**^	0.39^**^
9. Awareness	−0.06	0.09	0.08	0.04	0.15	0.02	0.09	0.13	1	−0.19^**^	0.13
10. PA	−0.00	−0.08	0.23^**^	0.41^**^	0.40^**^	0.40^**^	0.64^**^	−0.32^**^	0.00	1	−0.56^**^
11. NA	−0.09	−0.01	−0.33^**^	−0.40^**^	−0.42^**^	−0.43^**^	−0.54^**^	0.40^**^	−0.20^*^	−0.46^**^	1

*N* = 120; ^**^p ≤ 0.001, ^*^p < 0.01. NIS, Nature in self; PA, Positive affect; NA, Negative affect; Correlation coefficients above the diagonal indicate correlations between variables at T1, while correlation coefficients below the diagonal indicate correlations between variables at T2.

**Table 7:** ANOVA results for PPI and MBI groups.

Measures	PPI experiment group (*n* = 60)	MBI experiment group (*n* = 60)
Well-being	*F* (1,59) = 243.19, *p* < 0.001, η^2^ = 0.80	*F* (1,59) = 164.50, *p* < 0.001, η^2^ = 0.74
Gratitude	*F* (1,59) = 298.95, *p* < 0.001, η^2^ = 0.83	*F* (1,59) = 66.25, *p* < 0.001, η^2^ = 0.52
Nature in self	*F* (1,59) = 383.50, *p* < 0.001, η^2^ = 0.87	*F* (1,59) = 177.00, *p* < 0.001, η^2^ = 0.75
Connectivity	*F* (1,59) = 172.68, *p* < 0.001, η^2^ = 0.74	*F* (1,59) = 140.92, *p* < 0.001, η^2^ = 0.70
Resilience	*F* (1,59) = 28.82, *p* < 0.001, η^2^ = 0.33	*F* (1,59) = 48.03, *p* < 0.001, η^2^ = 0.45
Stress	*F* (1,59) = 46.90, *p* < 0.001, η^2^ = 0.44	*F* (1,59) = 118.14, *p* < 0.001, η^2^ = 0.67
Awareness	*F* (1,59) = 1.44, *p* > 0.005, η^2^ = 0.02	*F* (1,59) = 34.95, *p* < 0.001, η^2^ = 0.37
Positive affect	*F* (1,59) = 35.02, *p* < 0.001, η^2^ = 0.37	*F* (1,59) = 27.23, *p* < 0.001, η^2^ = 0.32
Negative affect	*F* (1,59) = 35.62, *p* < 0.001, η^2^ = 0.38	*F* (1,59) = 47.30, *p* < 0.001, η^2^ = 0.44

**Table 8:** *Post-hoc* analysis.

Measures	Group	Mean difference (SD)	*p* value	CI
Well-being	PPI and CTR	0.23 (0.04)^*^	0.000	0.13–0.33
	MBI and CTR	0.21 (0.04)^*^	0.000	0.11–0.31
	PPI and MBI	0.02 (0.04)	1.00	−0.08 – 0.12
Gratitude	PPI and CTR	0.38 (0.04)^*^	0.000	0.27–0.40
	MBI and CTR	0.30 (0.04)^*^	0.000	0.18–0.0.40
	PPI and MBI	0.08 (0.05)	0.25	−0.03 to 0.19
Nature in self	PPI and CTR	0.39 (0.04)^*^	0.000	0.3–0.48
	MBI and CTR	0.35 (0.04)^*^	0.000	0.26–0.44
	PPI and MBI	0.04 (0.04)	0.075	−0.05 to 0.13
Connectivity	PPI and CTR	0.32 (0.04)^*^	0.000	0.23–0.41
	MBI and CTR	0.26 (0.04)	0.000	0.17–0.35
	PPI and MBI	0.06 (0.04)	0.305	−0.02 to 0.15
Resilience	PPI and CTR	0.18 (0.04)^*^	0.000	0.09–0.27
	MBI and CTR	0.12 (0.04)^*^	0.004	0.03–0.21
	PPI and MBI	0.06(0.04)^*^	0.336	−0.03 to 0.15
Stress	PPI and CTR	−0.16 (0.06)^*^	0.018	−0.030 to −0.02
	MBI and CTR	−0.23 (0.06)^*^	0.000	−0.37 to −0.08
	PPI and MBI	0.06 (0.06)	0.813	−0.07 to 0.20
Awareness	PPI and CTR	0.09 (0.06)^*^	0.383	−0.05 to 0.23
	MBI and CTR	0.14 (0.06)	0.053	−0.20 to 0.09
	PPI and MBI	−0.05 (0.06)^*^	1.00	−0.00 to 0.30
Positive affect	PPI and CTR	0.15 (0.04)^*^	0.001	0.05–0.26
	MBI and CTR	0.09 (0.04)	0.115	−0.01 to 0.19
	PPI and MBI	0.06 (0.04)	0.386	−0.00 to 0.19
Negative affect	PPI and CTR	−0.15 (0.04)^*^	0.000	−0.02 to −0.07
	MBI and CTR	−0.12 (0.04)^*^	0.002	−0.21 to −0.04
	PPI and MBI	−0.03 (0.04)	1.00	−0.12 to 0.06

^**^p ≤ 0.001, ^*^p < 0.005.

Table 5 shows the means and standard deviations for all measures for PPI, MBI, and CTR groups at the two time points.

### Well-being

**Figure 1:**
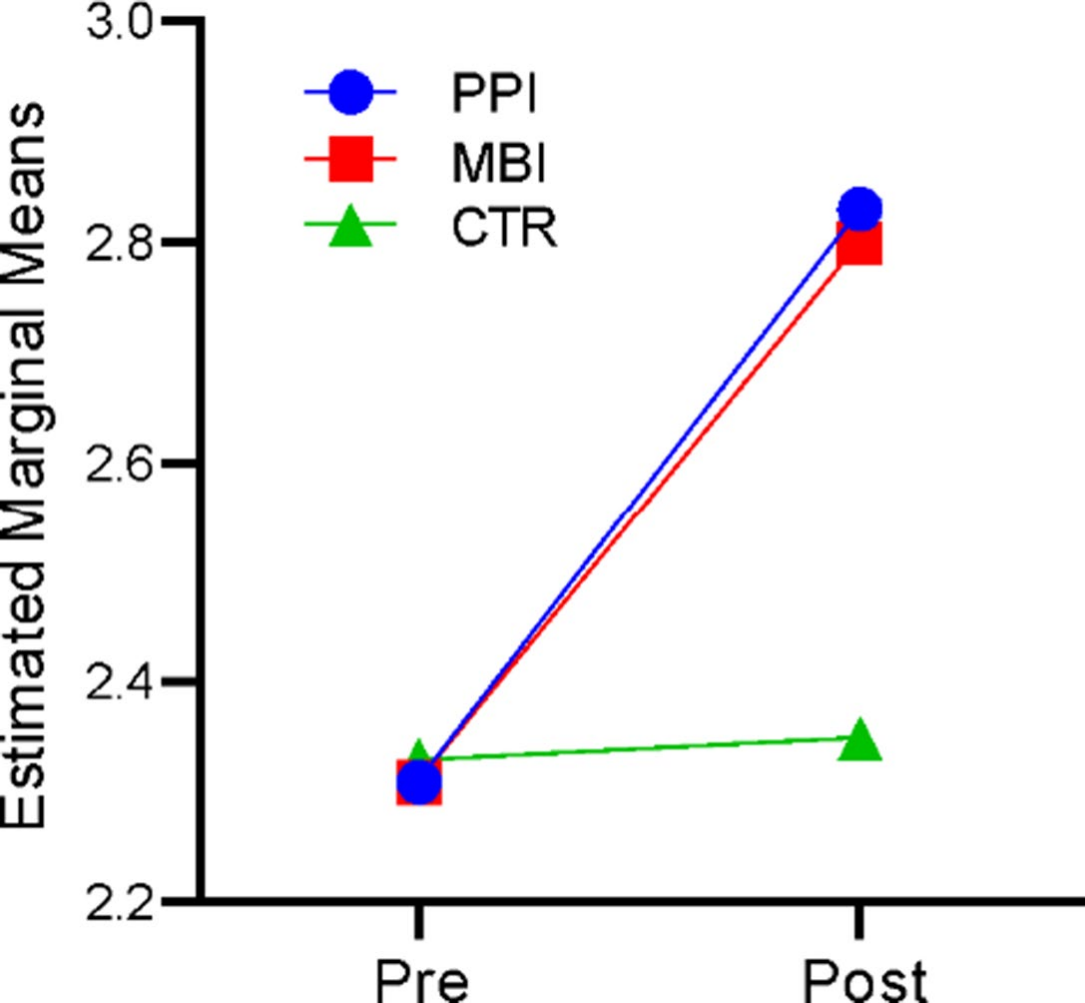
Pre–post-comparison of well-being.

A repeated-measures ANOVA revealed that time significantly affected well-being, *F* (1,177) = 340.262, *p* < 0.001, η^2^ = 0.658. There was also a significant time-by-group interaction, *F* (2,177) = 74.134, *p* < 0.001, η^2^ = 0.456. The results indicate that participants’ well-being levels differed significantly across the two time points (at baseline and post) and there was a significant difference in the well-being of the three groups (PPI vs. MBI vs. CTR). Figure 1 suggests that in the PPI and MBI groups, well-being steadily increased across the two time points, whereas the CTR group did not show a significant change from pre to post-time points.

### Gratitude

**Figure 2:**
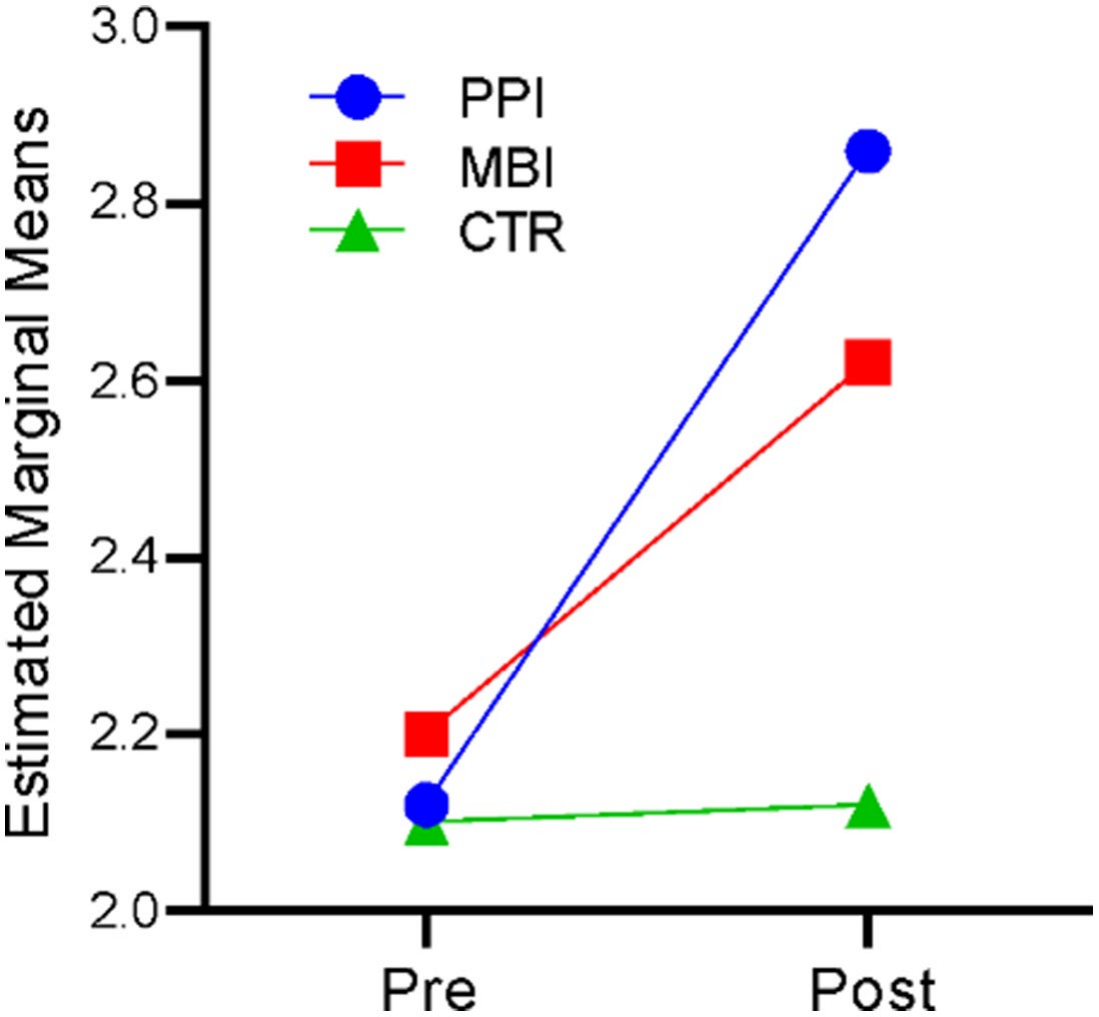
Pre–post-comparison of gratitude.

A repeated-measures ANOVA revealed that time significantly affected gratitude, *F*(1,177) = 297.076, *p* < 0.001, η^2^ = 0.627. There was also a significant time-by-group interaction, *F*(2,177) = 84.204, *p* < 0.001, η^2^ = 0.488. The results indicate that participants’ gratitude levels differed significantly across the two time points (at baseline and post), and there was a significant difference in gratitude among the three groups (PPI vs. MBI vs. CTR). Figure 2 suggests that the PPI and MBI groups showed a steady increase in gratitude across the two time points, whereas the CTR group did not significantly change from pre- to post-time points.

### Nature in self

**Figure 3:**
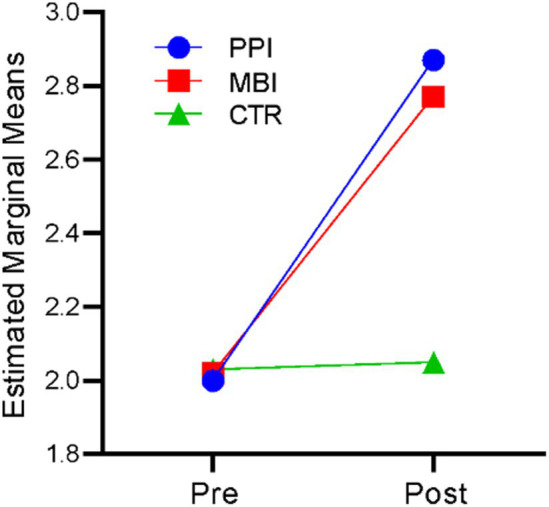
Pre–post-comparison of nature-in-self.

A repeated-measures ANOVA revealed that time significantly affected nature and self-connection, *F* (1,177) = 492.727, *p* < 0.001, η^2^ = 0.736. There was also a significant time-by-group interaction, *F*(2,177) = 117.641, *p* < 0.001, η^2^ = 0.571. The results indicate that participants’ nature and self-connection differed significantly across the two time points (at baseline and post), and there was a significant difference in nature and self-connection of the three groups (PPI vs. MBI vs. CTR). Figure 3 suggests that the PPI and MBI groups showed a steady increase in nature and self-connection across the two time points, whereas the CTR group did not significantly change from pre- to post-time points.

### Connectedness

**Figure 4:**
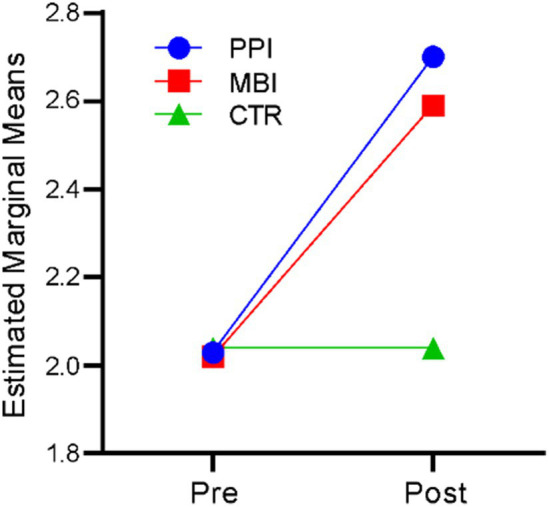
Pre–post-comparison of connectedness.

A repeated-measures ANOVA revealed that time significantly affected the sense of connectedness, *F*(1,177) = 313.471, *p* < 0.001, η^2^ = 0.639. There was also a significant time-by-group interaction, *F* (2,177) = 79.913, *p* < 0.001, η^2^ = 0.475. The results indicate that participants’ sense of connectedness differed significantly across the two time points (at baseline and post), and there was a significant difference in the sense of connectedness of the three groups (PPI vs. MBI vs. CTR). Figure 4 suggests that the PPI and MBI groups showed a steady increase in a sense of connectedness across the two time points, whereas the CTR group did not show a significant change from pre- to post-time points.

### Resilience

**Figure 5:**
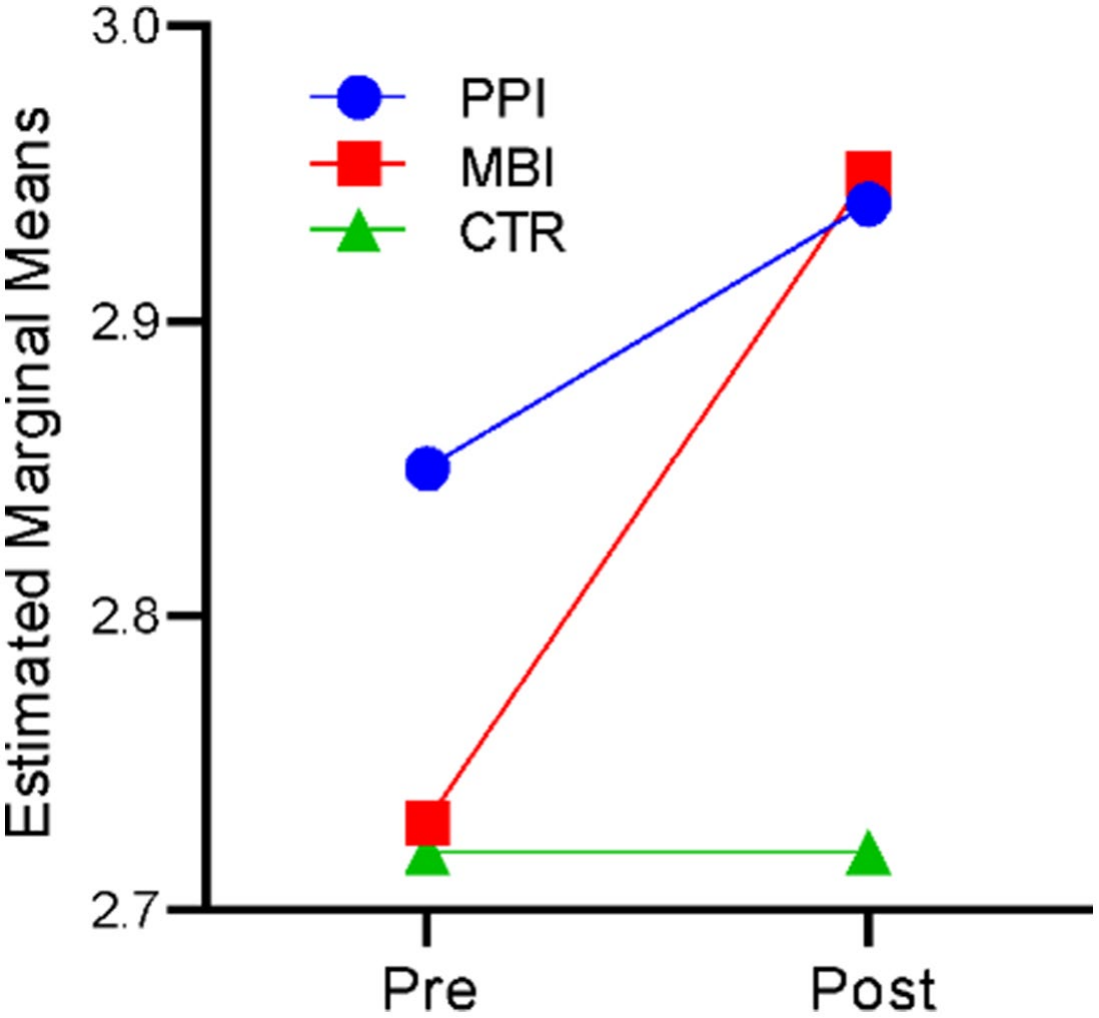
Pre–post-comparison of resilience.

A repeated-measures ANOVA revealed that time significantly affected resilience, *F*(2,117) = 27.065, *p* < 0.001, η^2^ = 0.234. There was also a significant time-by-group interaction, *F*(1,117) = 75.486, *p* < 0.001, η^2^ = 0.299. The results indicate that participants’ levels of resilience differed significantly across the two time points (at baseline and post), and there was a significant difference in resilience of the three groups (PPI vs. MBI vs. CTR). Figure 5 suggests that the PPI and MBI group showed a steady increase in resilience across the two time points, whereas the CTR group did not significantly change from pre- to post-time point.

### Stress

**Figure 6:**
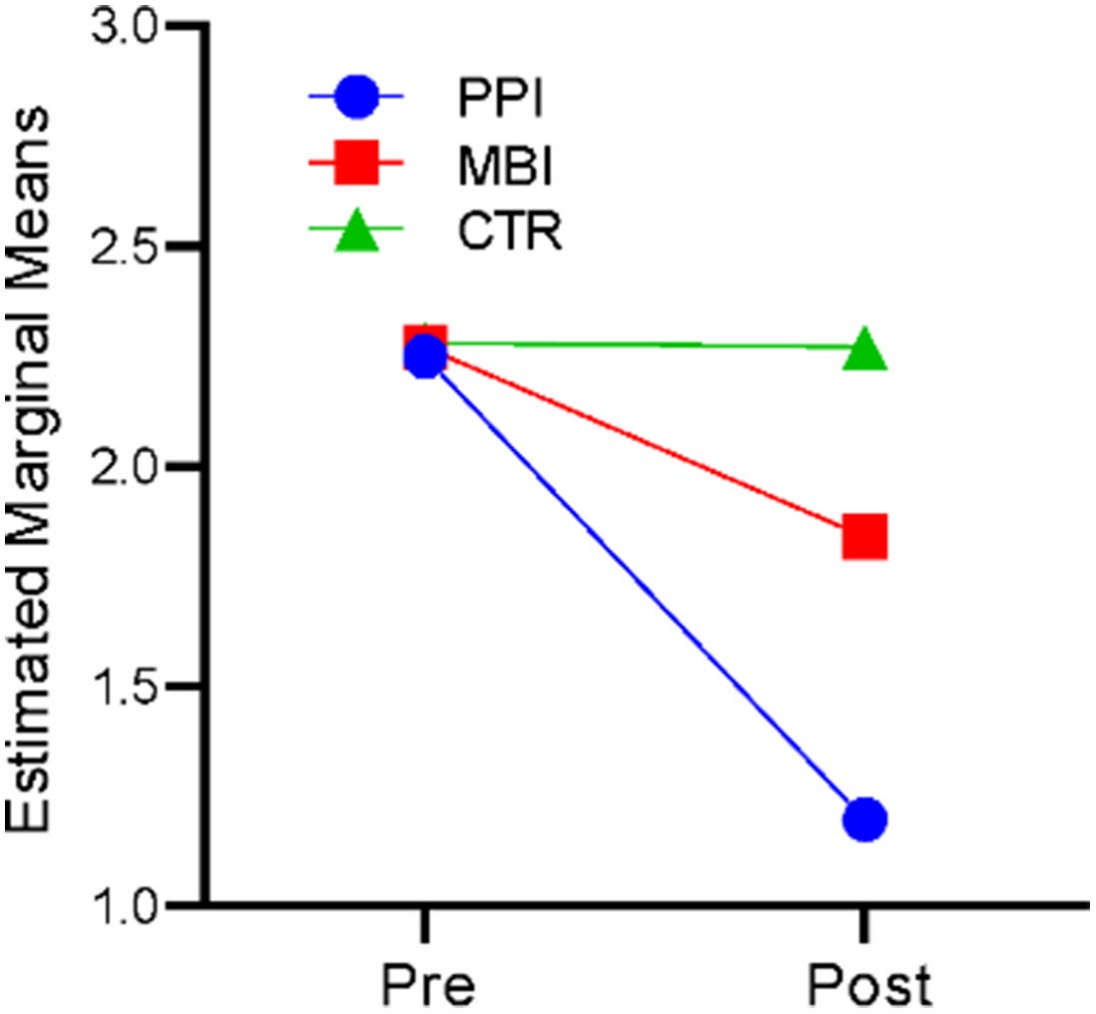
Pre–post-comparison of stress.

A repeated-measures ANOVA revealed that time significantly affected stress, *F* (1,177) = 157.542, *p* < 0.001, η^2^ = 0.471. There was also a significant time-by-group interaction, F (1,117) = 75.486, *p* < 0.001, η^2^ = 0.299. The results indicate that participants’ stress levels differed significantly across the two time points (at baseline and post), and there was a significant difference in stress levels of the three groups (PPI vs. MBI vs. CTR). Figure 6 suggests that in the PPI and MBI groups, stress levels decreased across the two time points, whereas the CTR group did not show a significant change from pre- to post-time points.

### Awareness

**Figure 7:**
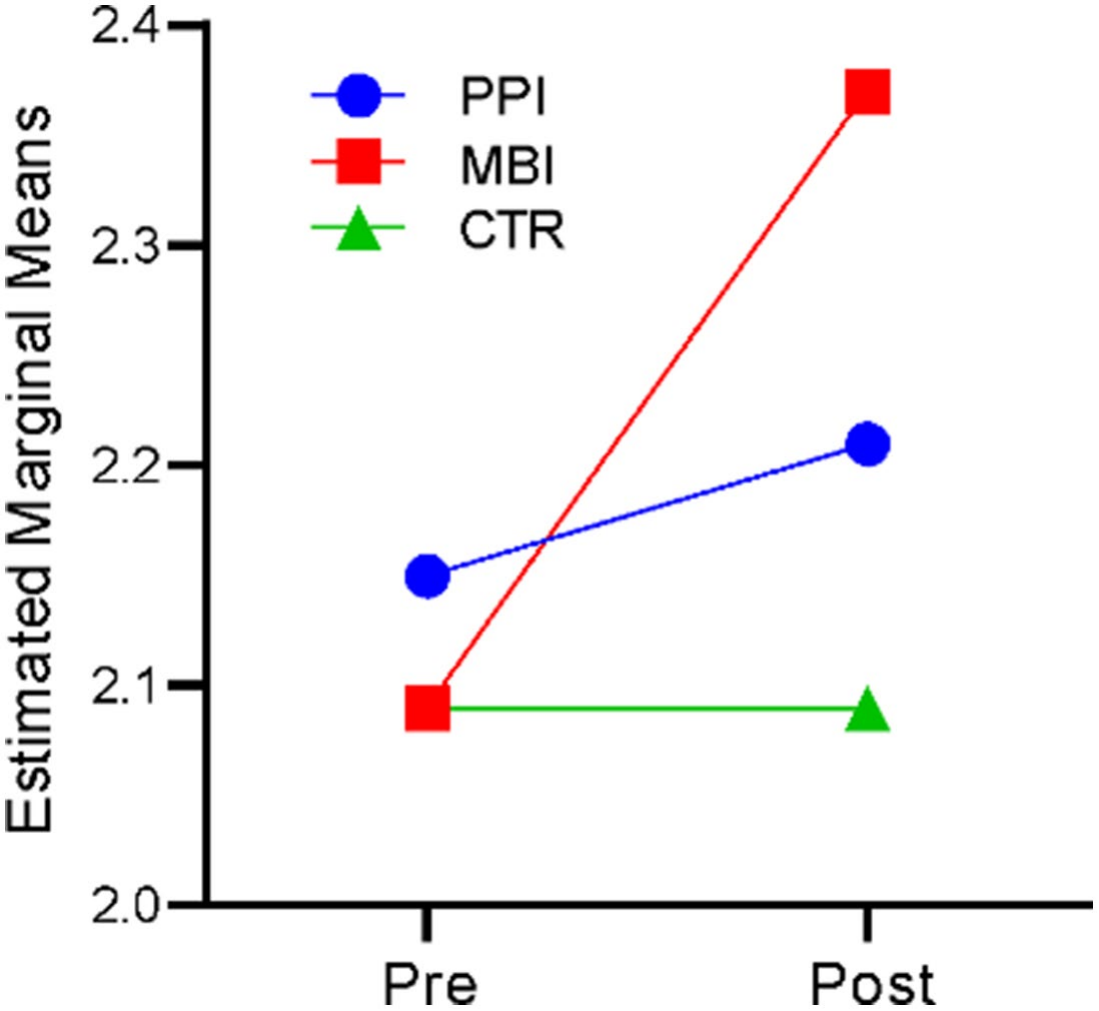
Pre–post-comparison of awareness.

A repeated-measures ANOVA revealed that time significantly affected awareness, *F*(1,177) = 25.529, *p* < 0.001, η^2^ = 0.126. There was also a significant time-by-group interaction, *F*(2,177) = 14.892, *p* < 0.001, η^2^ = 0.144. The results indicate that participants’ levels of awareness differed significantly across the two time points (at baseline and post), and there was a significant difference in awareness levels of the three groups (PPI vs. MBI vs. CTR). Figure 7 suggests that, in the PPI and MBI groups, a steady increase in awareness levels across the two time points was observed, whereas the CTR group did not show a significant change from pre- to post-time points.

### Positive affect

**Figure 8:**
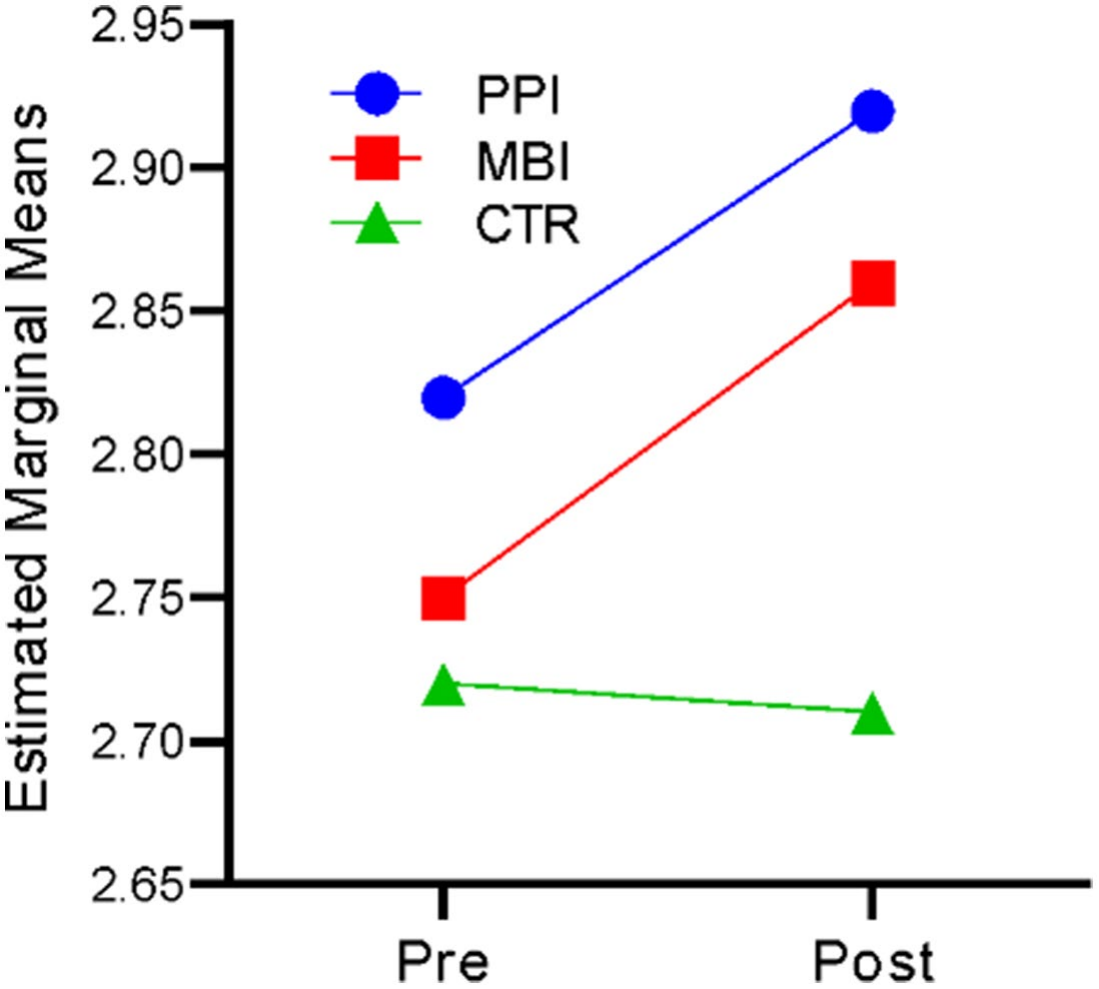
Pre–post-comparison of positive affect.

A repeated-measures ANOVA revealed that time significantly affected positive affect, *F*(1,177) = 45.87, *p* < 0.001, η^2^ = 0.206. There was also a significant time-by-group interaction, *F*(2,177) = 15.854, *p* < 0.001, η^2^ = 0.152. The results indicate that participants’ positive affect differed significantly across the two time points (at baseline and post), and there was a significant difference in the positive affect of the three groups (PPI vs. MBI vs. CTR). Figure 8 suggests that in the PPI and MBI groups, a steady increase in positive affect across the two time points was observed, whereas the CTR group did not significantly change from pre- to post-time points.

### Negative affect

**Figure 9:**
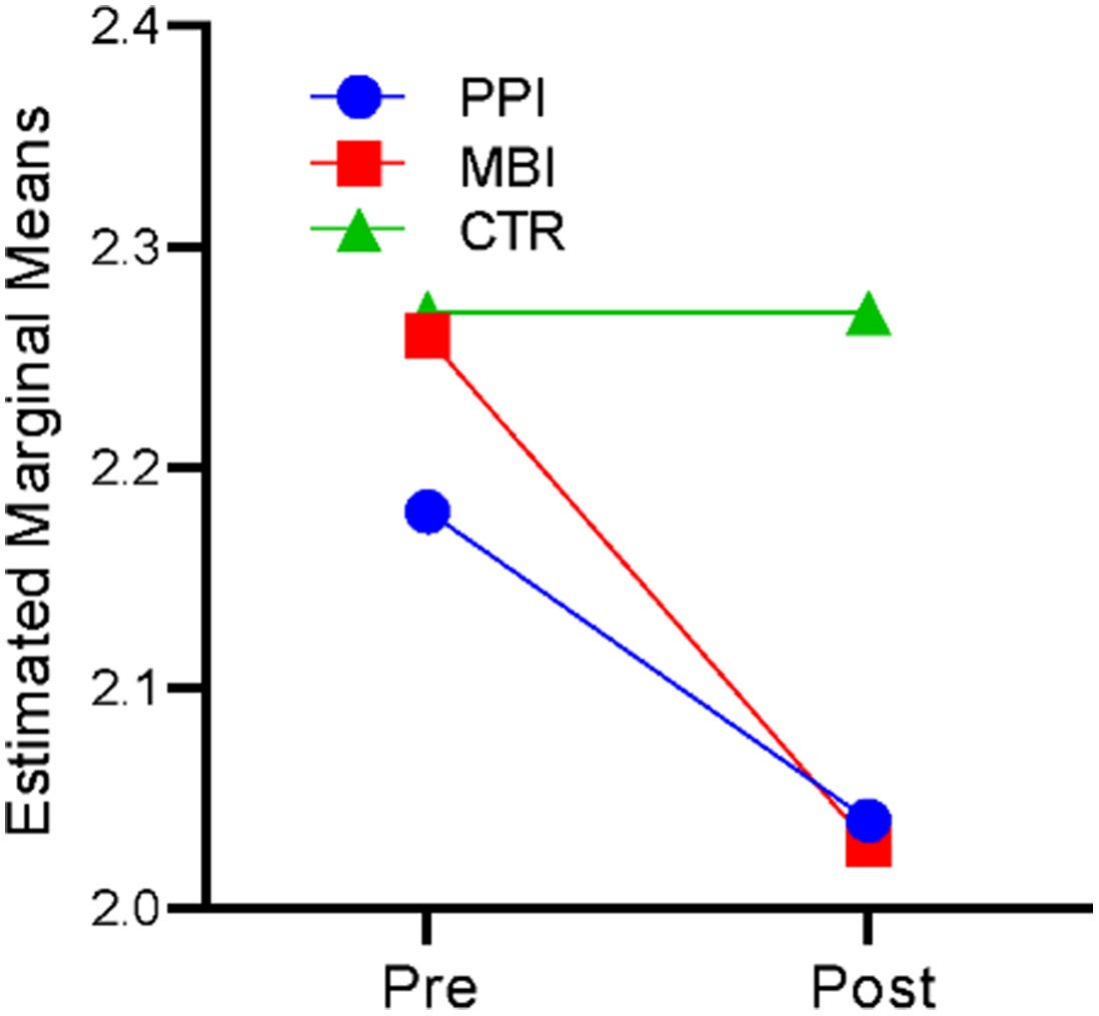
Pre–post-comparison of negative affect.

A repeated-measures ANOVA revealed that time significantly affected the negative affect, *F*(1,177) = 82.006, *p* < 0.001, η^2^ = 0.317. There was also a significant time-by-group interaction, *F*(2,177) = 24.207, *p* < 0.001, η^2^ = 0.215. The results indicate that participants’ negative affect differed significantly across the two time points (at baseline and post), and there was a significant difference in the negative affect of the three groups (PPI vs. MBI vs. CTR). Figure 9 suggests that in the PPI and MBI groups, the negative affect decreased across the two time points, whereas the CTR group did not show a significant change from pre- to post-time points.

## Discussion

This study aimed to develop, execute, and compare two well-being interventions, PPI and MBI, in natural settings for urban school students in India. Results suggest that both PPI and MBI demonstrate comparable effectiveness in improving student well-being when executed in nature. Both interventions displayed significant improvement in comparison with the control group in all variables, i.e., an increase in well-being, gratitude, nature in self, sense of connectedness, resilience, awareness, positive affect, and a decrease in stress and negative affect levels. This is in line with previous meta-analytic evidence on the effectiveness of MBIs in nature (34), MBIs in schools (28–32), and PPIs in a school setting (36, 37). These findings further add to the studies that we built upon, such as Pirchio et al. (88), who demonstrated positive outcomes of a school intervention focusing on contact with nature (on psycho-physical well-being, connectedness to nature, and prosocial behavior of students) in the intervention group than the control group as well as Choe and Sheffield (33), offering essential insights on the impact of natural surroundings in improving intervention efficacy.

Other studies conjoining mindfulness and positive psychology interventions have found similar effects. Ivtzan et al. (42) merged mindfulness and positive psychology elements in an 8-week positive mindfulness program (PMP). They found a significant improvement in all the dependent variables (happiness, stress, depression, gratitude, self-compassion, autonomy, efficacy, meaning, compassion for others, and appreciation) compared to the pre-test measurements and the control group. Compared with control group participants, an intervention containing work-related mindfulness exercises and positive psychology activities displayed increased mindfulness, positive affect, work engagement, hope, sleep quality, and reduced fatigue (43).

We also observed some intervention-specific effects. For instance, there were no significant differences in levels of awareness for participants of the PPI group in the pre- and post-condition. This might be because PPIs are traditionally designed to impact positive feelings, positive cognitions, or positive behavior (52) as opposed to directly influencing awareness, which is more so of a role of MBIs (89) as seen in the present article.

This was also the first study to test the effectiveness of a PPI in nature for urban school students in the Indian context. The results were consistent with previous literature on PPIs for school students (37, 38, 90). By highlighting the equivalency of these approaches, our research fills a gap in the nature-based well-being interventions in school by offering a more nuanced understanding of the potential interventions available for supporting and positively influencing the lives of urban youth and their connection with nature.

Our findings have practical implications for educators and practitioners working with urban youth populations. A recent meta-analysis of school-based multi-component PPIs stated that merging multi-component PPIs with other interventions bolsters the efficacy of the original PPI (91). By recognizing the viability of both PPI and MBI as effective interventions, decision-makers can combine the two interventions and informed choices when designing programs and allocating resources to support the development and well-being of urban school students. Schools can use these interventions outside of typical classrooms and investigate the use of natural areas. For example, outdoor places or nature reserves provide unique contexts for holistic growth, interpersonal interactions, and skill development among urban school students. As a result, schools should consider including and developing natural spaces within their premises to create conditions conducive to properly implementing PPI and MBI interventions.

The present study and its results have certain limitations. In this study, some factors may affect the generalizability of the findings. The sample of school students was derived from a single urban school in India, which restricts the extent to which the findings can be applied to a broader population. Additionally, the longitudinal aspect of the study was compromised as we were unable to track the sustainability of the results over time, which could have provided valuable insights. Furthermore, all interventions were conducted in a natural setting, thereby preventing the exploration of potential differences between the effects of PPI and MBIs in a classroom vs. a natural environment. Another limitation pertains to the shortage of personnel. Schoolteachers external to the study completed the data collection process, which introduces the possibility of bias and conformity pressure. These limitations should be considered when interpreting the results and considering the study’s implications.

In order to further advance the understanding of PPI and MBI conducted in a natural setting, further research in this area is needed. Implementing interventions of a longer duration is also suggested as previous literature indicates that extended interventions have a more enduring impact. This could provide valuable insights into the sustained effects of nature-based interventions on the well-being of students. Additionally, incorporating an active control group in future studies would enhance the rigor of the research design. This would allow for a more accurate comparison between the effects of PPI or MBI with other interventions or activities, providing a clearer understanding of the specific benefits derived from the nature-based intervention. Furthermore, it is recommended to include control measures such as weather and humidity ratings as well as monitoring the consumption of mood-altering substances such as coffee, supplements, energy drinks, and chocolate. These control measures might help identify potential confounding factors that could influence the outcomes of the intervention.

To enhance the effectiveness of the interventions and engagement of urban school students, including homework assignments could be considered. Finally, to gain deeper insights into the experiences and perceptions of urban school students regarding future nature-based interventions, conducting qualitative research methods such as focus groups with students would be beneficial. This would provide a platform for students to express their perspectives, suggestions, and ideas, aiding in developing more engaging and tailored interventions for this specific demographic.

Our findings demonstrate that PPI and MBI interventions can significantly improve student well-being when administered in nature. The study provides valuable insights for school authorities, policymakers, and urban planners to include natural settings in school premises and offer well-being interventions for students to connect with nature consciously. Incorporating nature-based modalities into educational settings may foster individual well-being and broader social and environmental health.

## Data availability statement

The raw data supporting the conclusions of this article will be made available by the authors, without undue reservation.

## Ethics statement

The studies involving humans were approved by the Institutional Review Board of the Indian Institute of Management Indore (reference number IRB/03/2022-23/HSS). The studies were conducted in accordance with the local legislation and institutional requirements. The participants provided their written informed consent to participate in this study.

## Author contributions

RC: Conceptualization, Data curation, Formal analysis, Investigation, Methodology, Project administration, Resources, Software, Supervision, Validation, Writing – original draft, Writing – review & editing. NH: Conceptualization, Methodology, Resources, Visualization, Writing – original draft, Writing – review & editing.
